# Gastrointestinal Symptoms and FODMAP Intake of Aged-Care Residents from Christchurch, New Zealand

**DOI:** 10.3390/nu9101083

**Published:** 2017-09-29

**Authors:** Wathsala S. Nanayakkara, Richard B. Gearry, Jane G. Muir, Leigh O’Brien, Tim J. Wilkinson, Jonathan A. Williman, Paula M. L. Skidmore

**Affiliations:** 1 Department of Human Nutrition, University of Otago, P.O. Box 56, Dunedin 9054, New Zealand; wathsala.k11@gmail.com (W.S.N.); leigh@canterburydietitians.co.nz (L.O.); 2 Department of Medicine, University of Otago, P.O. Box 4345, Christchurch 8140, New Zealand; Richard.Gearry@cdhb.health.nz (R.B.G.); tim.wilkinson@otago.ac.nz (T.J.W.); 3 Department of Gastroenterology, Monash University, 99 Commercial Rd, Melbourne VIC 3004, Australia; jane.muir@monash.edu; 4 Department of Population Health, University of Otago, P.O. Box 4345, Christchurch 8140, New Zealand; jonathan.williman@otago.ac.nz

**Keywords:** FODMAPs, gastrointestinal symptom, irritable bowel syndrome, older people

## Abstract

Studies on fermentable oligo-, di-, and monosaccharides as well as polyols (FODMAPs) intake in older adults are lacking. This study investigated the relationship between gastrointestinal (GI) symptoms and FODMAPs in aged care residents. The Gastrointestinal Symptom Rating Score questionnaire modified for patients with IBS (GSRS-IBS) was used to identify participants with IBS-like symptoms. Dietary intake was assessed for a subgroup of participants with highest total GSRS-IBS score (symptomatic cases) and age, sex, and level of care matched participants with low total GSRS-IBS score (asymptomatic controls). Seventy-four participants with a mean (SD) age of 86 (6.6) years completed the GSRS-IBS questionnaire and dietary data were collected using food diaries from a subsample of 27 symptomatic and 27 asymptomatic participants. The study found many older adults with functional gut symptoms. There were no differences between the groups for FODMAP intake and no significant relationship was found between FODMAP intake and total GSRS-IBS score. Lactose from milk and milk-based desserts was the biggest FODMAP contributor (16 g/day) and a significant relationship between total FODMAP intake and diarrhoea was found. A larger study sample in future studies is required to better capture symptomatic cases and manipulation of dietary FODMAPs may assist with the management of IBS in the elderly.

## 1. Introduction

Consistent with other developed countries, the New Zealand population is aging. The number of people aged 65 years and above has increased by 3.6% from mid-2015 to mid-2016 and now represents 15% of the total population [1]. This phenomenon is partly due to the rising life expectancy and results from many causes including advances in medical technology and sanitation, typical of a country that has undergone an “epidemiologic transition” [2]. In New Zealand, the life expectancy at birth has increased from 76.3 years (male) and 81.1 years (female) in 2001 to 79.5 and 83.2 years for males and females, respectively, in 2013 [3]. Such population growth has a huge impact on the cost of health as well as disability services, particularly the demand for long-term care facilities [4]. 

Aging is also associated with physiological changes including changes in the gastrointestinal (GI) tract [5,6] with an increased prevalence of GI disorders [7,8], which, in turn, can affect the nutritional status of older adults [9]. Irritable bowel syndrome (IBS) is a chronic disorder characterised by symptoms of abdominal discomfort or pain, bloating and altered bowel habits [10]. While there is no associated mortality [11], there is significantly impaired quality of life [12,13]. The prevalence of IBS is estimated to be 10–15% in Western populations and is more common in women [14]. Irritable bowel syndrome is predominantly seen in younger people, particularly those under 30 [15] and prevalence is 25% lower in patients aged over 50 years compared to younger patients [16]. Few studies have assessed the prevalence of IBS in the elderly, however, it is believed that as many as 22% of older people may have symptoms suggestive of IBS [17]. Many patients with IBS will significantly restrict food intake in an attempt to control their symptoms [18] which, in combination with reduced intake of food and drink often seen in aging, may lead to nutritional inadequacy [9]. 

Troublesome symptoms, in particular diarrhoea and faecal incontinence, can be both psychologically and socially debilitating and have a huge impact on frail elderly people. For instance, long-term faecal incontinence was found to be associated with increased mortality in long-term aged care residents [19] as faecal incontinence is one of many factors associated with frailty in older adults. Therefore, it is vital that effective treatment is available to improve quality of life in affected elderly people as well as reducing healthcare costs associated with faecal incontinence and diarrhoea. The low FODMAP (Fermentable Oligosaccharides, Disaccharides, Monosaccharides and Polyols) diet has been shown to significantly improve functional gut symptoms in adults (mean age 35–43 years) with IBS [20,21,22]. However, neither the FODMAP composition of the diet of older people nor the efficacy of the low FODMAP diet in the elderly has been studied previously. 

Therefore, the aims of this paper are to assess the frequency of GI symptoms, food sources and intake of FODMAPs, and to investigate the relationship between FODMAPs and IBS symptoms in older adults living in a residential care facility. 

## 2. Materials and Methods

This study is an exploratory cross-sectional observational study based at a single residential care facility in Christchurch, New Zealand. A retirement village of suitable size that was geographically near to the university was selected to provide a convenience sample. This retirement village provided two levels of care where residents were provided with all food and drink—rest home (RH) and hospital-level (H). In New Zealand, RH care is provided to support or to promote independence of frail people while H care is provided for those who require 24 h nursing care [23]. Those receiving respite care, or who were temporary residents, those receiving enteral feeding and those considered to be end-of-life were ineligible to take part. It was estimated that a sample size of 26 participants per group (case and controls) would provide 80% power (α = 0.05, two-sided) to detect large effect sizes (0.8 or higher). 

Written consent was obtained from all participants including consent from the next of kin or the legal representative for those participants with recognised cognitive impairment. Recruitment and data collection were conducted between July 2015 and January 2016. Participant consent and clinical information including the GI symptom questionnaire detailed below were collected in July/August 2015 and the dietary intake was assessed from November 2015 to January 2016. Ethics approval was obtained through The University of Otago Human Ethics (Health) Committee (H15/053). 

The validated Gastrointestinal Symptom Rating Scale modified for use in patients with IBS (GSRS-IBS) questionnaire [24] was used to help identify participants with IBS-like symptoms. The questionnaire contains 13 questions on GI symptoms which can be grouped into five domains; abdominal pain, bloating, constipation, diarrhoea and satiety. Responses of perceived severity of each symptom experienced in the past week are given in a 1 to 7 Likert scale where 1 is “no discomfort” and 7 is “very severe discomfort”. Possible total scores range from 13 to 91. The participants were interviewed face-to-face by the researcher using the GSRS-IBS questionnaire. A registered nurse verified responses of participants with mild short-term memory loss. For those participants with dementia, a nurse familiar with the participant completed the questionnaire. Bowel charts were cross-referenced for frequency and types of bowel movements for the week. Currently there is no standardised “cut-off” level for identifying people with IBS using the GSRS-IBS. Therefore, participants were ranked according to total GSRS-IBS score (sum of responses to the 13 questions) from highest to lowest score and the 30 participants with the highest GSRS-IBS score were selected as the “symptomatic” group. These 30 participants were then age (±5 years), sex and level of care (either RH or H level) matched to 30 participants with lower total GSRS-IBS score as the “asymptomatic” group. Demographic data (date of birth, gender and ethnicity) and health information on weight, height, body mass index (BMI), medical history, prescribed medications and functional status (continence, mobility and eating) were also collected from individual clinical notes of the participants. 

A combination of weighed/estimated/recalled three-day diet record (3DDR) was used to assess dietary intake for all participants. The combination was necessary to avoid delaying meal services and to minimise work load for the researcher at this busy residential care facility where residents consumed meals at multiple locations including the dining halls, in their individual rooms, and lounges. The food items for small and standard serving sizes were weighed for each meal provided by the facility using a Salter Model 1010 electronic kitchen scale with accuracy to within ±1 g (Salter Housewares Ltd., Kent, UK, range 1–2000 g). Each participant’s meal was photographed before and after consumption and the proportions of each food or beverage item consumed were estimated using these photographs. The 3DDR were collected for 60 selected participants from the symptomatic and asymptomatic groups for three main meals, snacks (morning and afternoon tea and supper), beverages and food and alcohol consumed during “Happy Hour”. The recordings consisted of non-consecutive two weekdays and a weekend day. “Meals consumed” included all foods and beverages actually consumed by the participants throughout the day including additional food and drinks brought in by visitors. Any foods or beverages consumed overnight following supper the night before were asked about at breakfast from the participants who could recall. 

The four week cycle menu with recipes were obtained from the facility and nutrient intake of these was analysed using FoodWorks 8 Professional Edition (version 8.0.3553, 2015, Xyris Software, Highgate Hill, Australia). The FODMAP database from Monash University, Department of Gastroenterology, Melbourne, Australia was used to analyse relevant food within each recipe for the FODMAPs. Appropriate substitutions were made for those foods that were not on the FODMAPs database such as canned fruit, different varieties of jams and biscuits. The main sources of individual FODMAPs in the participants’ diet was determined by per cent contribution of food or beverage item consumed to total oligosaccharides, polyols, fructose in excess of glucose and lactose. 

Participants who ate out and missed one or more main meals (either breakfast, lunch or dinner) from the facility were excluded from the final dietary analysis. A *p*-value of < 0.05 was considered statistically significant. Differences between gender or levels of care (RH and H) for total GSRS-IBS score were investigated using Kruskal–Wallis tests. Independent T tests were used to investigate differences in nutrient intake between the symptomatic and asymptomatic groups. Linear regression was used to determine the relationship between IBS symptom scores and total FODMAP intake, and logistic regression adjusted for age, gender and levels of care to investigate difference in total FODMAP intake by symptomatic versus asymptomatic participants. All statistical analyses were performed using RStudio (version 0.99.903, 2009–2016, RStudio, Boston, MA, USA). 

## 3. Results

One hundred and twenty-eight RH and H residents aged over 65 years old were living at the facility. Six of these did not meet the inclusion criteria (respite care, end-of-life, and enteral feeding) and four died and two relocated to another facility during the data collection period. Therefore, 116 eligible RH and H care residents were invited to take part in the study. Of these 74 (64%) completed the GSRS-IBS questionnaire. Demographic characteristics of these 74 participants and 30 participants each selected for symptomatic and asymptomatic groups are summarised in Table 1. 

The majority of the participants were females (72%) and all 74 participants were Caucasians. Thirty-four (46%) of these participants were in RH care and the remaining 40 (54%) were in H level of care. None of the participants were medically diagnosed with IBS, however, 20% had a diagnosis of lower GI disorders (Table S1). These lower GI disorders included constipation, diverticular disease, small bowel obstruction, bowel resection and one participant with coeliac disease. Just over half of the participants were on regular medications daily for the treatment of constipation and 69 (93%) were prescribed laxatives as needed. Eleven percent of participants were on diarrhoea treatment and 20% had faecal incontinence. Twenty-five (34%) had recognised cognitive impairment and, of them, eight (11%) had a diagnosis of dementia. There was no difference between the use of lactulose between the two groups. 

The median (range) for total GSRS-IBS scores was 16.5 (13–40) and the interquartile range was 14 and 21 for total GSRS-IBS score. There were no significant differences between gender or levels of care (RH and H) for total GSRS-IBS score. The cut-off for total GSRS-IBS score to produce the symptomatic group was 17. In each group, 17 (57%) participants were H level and 13 (43%) were RH level of care. The median total GSRS-IBS score (range) for all 13 questions were 21.5 (17–40) and 14.5 (13–17) for symptomatic and asymptomatic group respectively. The average number of IBS symptoms reported by the symptomatic group was 5 (range 2–9 symptoms). In the asymptomatic group, the average number was 1 (range 0–3). Table 2 shows the differences between the groups for reporting any or severe symptom for each GSRS-IBS question under the five domains. Table S2 include responses to each question by the participants. At least half of the participants in the symptomatic group reported symptoms related to all five GSRS-IBS subdomains, and about one third of these participants reported moderate to severe discomforts for abdominal pain and diarrhoea. A much lower proportions of asymptomatic participants reported any symptoms (less than 30%) for the five subdomains. Overall, bloating-related followed by diarrhoea- and constipation-related symptoms were most commonly reported by the participants. 

Of the 60 participants allocated to the symptomatic and asymptomatic groups, 54 (27 from each group) consumed all main meals at the facility, while five participants ate out at least one main meal and one participant had relocated to another facility before dietary data collection was completed. The average age of those participants who ate all main meals at the facility was 87 ± 6.45 years (compared to 82 ± 5.89 years of five participants who did not), however the other characteristics were similar including the median total GSRS-IBS scores (Table 3). Table 4 compares the average macronutrients and FODMAPs consumed by symptomatic and asymptomatic groups. There were no significant differences in intakes between the two groups. 

The major food sources of FODMAPs consumed by the study participants are summarised in Table 5. A large amount of oligosaccharides were obtained from the wheat-based products such as bread, cakes and biscuits. Sixteen residents were on oral nutritional supplements with some containing fructo- and galacto-oligosaccharides, which contributed (17%) towards the total oligosaccharides consumed by the 54 residents. Sources of polyols were mostly from fruit such as canned stewed prunes (28%), canned pears (26%) and canned peaches (9%). Fructose in excess of glucose was also mostly from canned fruit such as pears (32%). A large proportion of lactose came from cow’s milk added to beverages and cereals (39%) and used to cook porridge (27%). 

**Figure 1:**
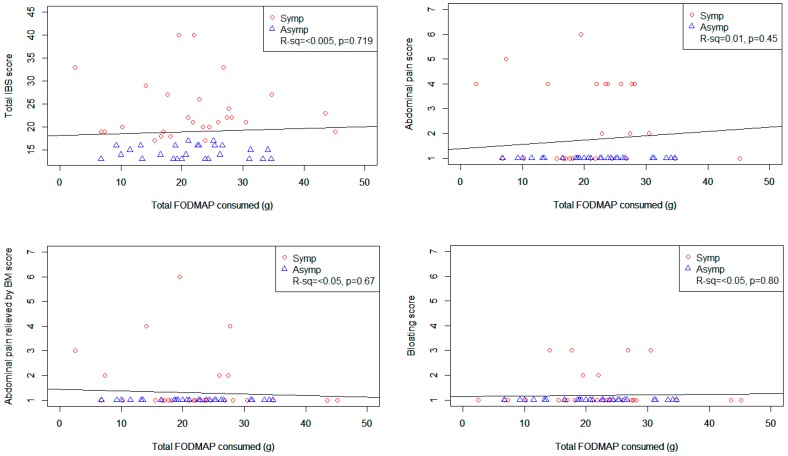

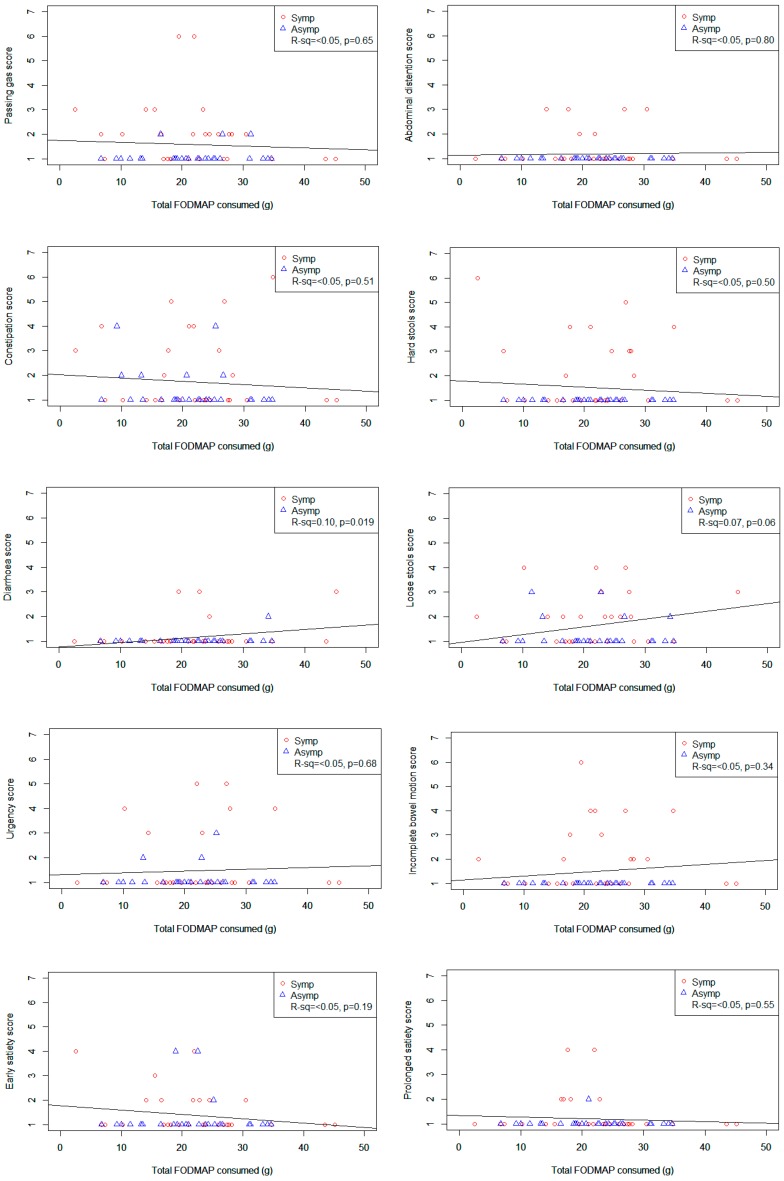
Scatter plots with linear regressions of total FODMAP intake including lactose and various IBS symptom scores. Presented *R*-squared and *p*-values indicate the significance of the association between total FODMAP intake and IBS symptom score.

Figure 1 shows scatter plots of various GSRS-IBS symptom scores plotted against total FODMAP intake. The only statistically significant relationship was seen between total FODMAP intake and diarrhoea (*r*^2^ = 0.1, *p* = 0.019) which showed increasing discomfort with increasing intakes of total FODMAPs. However, when lactose was removed from the total FODMAP, there was no relationship between total FODMAP intake and total or individual IBS symptoms (see Figure S1). 

The results from the logistic regression analyses of total GSRS-IBS score and total FODMAP including lactose intake adjusted for age, gender and level of care did not show a significant relationship (*p* = 0.73). Similarly, there was no relationship between GSRS-IBS score and total FODMAP excluding lactose intake (*p* = 0.86). However, there was a significant association between age and total FODMAP intake (for every 10 year increase in age, participants consumed 1.53 grams less total FODMAPS, 95% CI = 0.61 to 2.46, *p* = 0.002). Similarly, a significant negative relationships between age and total energy intake (estimate −900 kJ per 10 years; 95% CI −191 to −1610; *p* = 0.014) was also seen. 

## 4. Discussion

To our knowledge, currently there are no published data on the frequency of GI symptoms of New Zealand adults over the age of 65 years. We believe our study is the first to describe this using the validated GSRS-IBS questionnaire as most studies exclude older participants. To our knowledge, this study is also the first to describe FODMAP intake in older adults living in a long-term aged care facility and to observe the entire diet of participants in their own environment for FODMAP intake. This study has shown that at least half the study participants experienced functional gut symptoms, particularly diarrhoea-, and bloating-related symptoms.

We found that lactose was a large contributor to the total FODMAPs intake, which contrast with a typical Australian diet where fructose in excess of glucose, oligosaccharides and polyols contribute more towards total FODMAPs than lactose [20,25]. Although we did not assess the rate of lactose intolerance in our study, Stefano and his colleagues [26] found a high prevalence (83%) of lactose malabsorption in the adults aged 75 years and over, however, only 48% were found to be lactose intolerant with complaints of abdominal symptoms following consumption of milk and dairy products. Nonetheless, this prevalence of lactose intolerance is much higher compared to younger adults where typically the prevalence is less than 30% in Europeans [27]. Therefore, offering lactose-free alternatives such as calcium fortified rice or almond milk might be an easy food alteration to trial in symptomatic participants. Future studies should also investigate the prevalence of lactose intolerance in this elderly population using lactose intolerance tests and breath hydrogen tests to confirm whether there would be a benefit to substituting cow’s milk in the diet. 

Future studies exploring the effect of reducing FODMAPs to control GI symptoms in elderly people are required. In the elderly population, exploring the use of a more simplified version of low FODMAP diet (i.e., a “modified” low FODMAP diet) may be more appropriate. It is noteworthy some FODMAPs (e.g., oligosaccharides-fructans and galacto-oligosaccharides) are prebiotic and may have important implications for encouraging the growth of bacteria with “putative” health benefits. For this reason, these types of FODMAPs should be reintroduced into the diet to a level that can be tolerated. It is not advised that a strict low FODMAP diet is followed over long-term. 

We did not find a significant association between total FODMAP intake and GI symptoms of IBS. This may be a true finding where, in an elderly population, there is no association between FODMAP intake and GI symptoms. However, a more likely explanation is that the amount and nature of FODMAPs causing symptoms may vary significantly between individuals and, therefore, a linear association is unlikely to be identified. A small significant relationship was found between total FODMAP intake and diarrhoea when the total FODMAPs included lactose. Although a large proportion of the participants complained of diarrhoea-related symptoms, a majority (35%) complained loose stools rather than diarrhoea or “frequent bowel movements” (8%) as asked on the GSRS-IBS. Therefore, this result would need to be confirmed in a larger study. 

There may be study design limitations that contributed to observing no significant relationship. Firstly, the study may have been under-powered to show a significant effect. Secondly, it is possible that rates of IBS are much lower among long-term aged care residents. Thirdly, the prescription of multiple medications may have masked or managed symptoms of IBS, particularly as we note that majority of participants (93%) had laxatives prescribed for use if needed, and over half used these on daily basis. Fourthly, the selection of cases may not have been sufficiently rigorous. It was assumed that the presence of GI symptoms in this population may reflect functional GI symptoms consistent with IBS, but the patients may have been suffering from other causes of GI symptoms (such as those due to comorbidities or their treatments, medications, previous GI surgery, effects of immobility, etc.). In future, inclusion criteria may need to be restricted to those participants on fewer medications and to exclude those with dementia to maintain accuracy of the GI symptoms data. We have excluded those residents who missed at least one main meal from the facility to maintain accuracy of the dietary data collected, however, there was no outstanding differences between those who ate in the facility and the handful of participants who ate out, it is unlikely that this has led to selection bias.

As only one residential care facility was included in the study, the results cannot be generalised to all older adults living in long-term care facilities. The demographics, specifically with regards to ethnicity, where all of the participants were Caucasian in this study is not representative of the New Zealand population. Other limitations include incomplete FODMAP analysis due to some food items not yet being analysed (such as canned fruit which needed to be substituted with a proportion of fresh fruit). 

Finally, with regards to the validated GSRS-IBS questionnaire that was used, a major limitation of this tool is the lack of standardised cut-off for determining patients with IBS. Additionally, some of the language was less than optimal for the study population and may have led to confusion for some participants. For example, the word “bothered” could be changed. Furthermore, while the GSRS-IBS categorises “sensation of incomplete bowel evacuation” as part of the “diarrhoea” subdomain, it could also result from constipation. 

The strengths of this study are as follows. One researcher conducted all data collection and data entry to remove inter-investigator variation. The dietary intake was also collected using a modified weighed 3DDR to avoid errors of reporting by the participants or the staff, particularly under-reporting. Furthermore, actual participants’ intakes were observed and recipes collected from the facility allowing better quality of data being entered for nutrient analysis.

## 5. Future Directions 

The current study did not identify a clear cut GSRS-IBS score between the symptomatic and asymptomatic groups. A much larger study in multiple aged-care facilities is needed to produce more representative results of long-term aged care residents. As there is no standardised cut-off for GSRS-IBS, future studies looking at prevalence of IBS among older adults may benefit from using the diagnostic criteria such as the Rome IV criteria for IBS, however, this requires a physician or a medical professional qualified to diagnose patients. Special care is needed due to the age group being investigated, to rule out other serious diagnosis such as colorectal cancer. We also suggest developing a questionnaire that is better suited to older people for assessing GI symptoms. Our results indicated that both symptomatic and asymptomatic groups were consuming similar amount of FODMAPs. Future research in this area could explore the application of a “modified” low FODMAP dietary approach in symptomatic residents. 

## Figures and Tables

**Table 1:** Baseline demographic of 74 participants who completed GSRS-IBS questionnaire and 30 participants each selected for symptomatic and asymptomatic groups.

Characteristic	74 Participants	Symptomatic Group (*n* = 30)	Asymptomatic Group (*n* = 30)
Age, mean ± SD in years	86 ± 6.55	86 ± 6.36	87 ± 6.83
Gender			
Men	21 (28%)	7 (23%)	7 (23%)
Women	53 (72%)	23 (77%)	23 (77%)
Level of care			
Rest Home	34 (46%)	13 (43%)	13 (43%)
Hospital	40 (54%)	17 (57%)	17 (57%)
Ethnicity			
NZ European	66 (89%)	25 (83%)	28 (93%)
Other	8 (11%)	5 (17%)	2 (7%)
Body mass index			
Average (kg/m^2^)	23.9	24.7	22.8

**Table 2:** Number of symptomatic and asymptomatic participants reporting any discomfort and moderate to severe discomfort in the five subdomain of the GSRS-IBS questionnaire.

	Symptomatic Group (*n* = 30)		Asymptomatic Group (*n* = 30)		Overall (*n* = 60)
GSRS-IBS Subdomains	Score ≥ 2 (Any Discomfort), *n*	Score ≥ 4 (Moderate to Severe Discomfort), *n*	Score ≥ 2 (Any Discomfort), *n*	Score ≥ 4 (Moderate to Severe Discomfort), *n*	Score ≥ 2 (Any Discomfort), *n*
Pain (Q1 or 2)	15 (50%)	11 (37%)	1 (3%)	0	16 (27%)
Bloating (Q3, 4 or 13)	24 (80%)	3 (10%)	7 (23%)	1 (3%)	31 (52%)
Constipation (Q5 or 8)	17 (57%)	8 (27%)	8 (27%)	3 (10%)	25 (42%)
Diarrhoea (Q6, 7, 9 or 10)	24 (80%)	10 (33%)	6 (20%)	0	30 (50%)
Satiety (Q11 or 12)	15 (50%)	3 (10%)	5 (17%)	2 (7%)	20 (33%)

Abbreviations: GSRS-IBS, IBS specific Gastrointestinal Symptom Rating Scale. Notes: *n* (%) for each subdomains was determined by adding the number of participants who scored ≥2 or ≥4 for any of the relevant questions under each subdomain.

**Table 3:** Demographics and median total GSRS-IBS scores of 54 participants who consumed all three main meals at the facility and 5 participants who ate out at least one main meal.

Characteristic	Consumed All Main Meals Provided (*n* = 54)	Ate Out at Least One Main Meal (*n* = 5)
Age, mean ± SD in years	87 ± 6.45	82 ± 5.89
Gender		
Men	12 (22%)	1 (20%)
Women	42 (78%)	4 (80%)
Level of care		
Rest Home	24 (44%)	2 (40%)
Hospital	30 (56%)	3 (60%)
Ethnicity		
NZ European	49 (91%)	4 (80%)
Other	5 (9%)	1 (20%)
Average body mass index (kg/m^2^)	23.7	24.5
Median total GSRS-IBS score	17	17

**Table 4:** Average daily intake of macronutrients and FODMAPs consumed by symptomatic and asymptomatic groups.

Nutrients	Symptomatic Group (*n* = 27) Mean (SD)	Asymptomatic Group (*n* = 27) Mean (SD)	95% CI	*p*-Value ^a^
Energy (kJ)	6516 (1752)	6487 (1769)	−933 to 990	0.95
Protein (g)	50.0 (15.1)	50.2 (15.3)	−8.46 to 8.13	0.97
Total fat (g)	66.7 (20.46)	65.4 (21.92)	−10.3 to 12.9	0.82
Carbohydrate (g)	178 (50.7)	180 (49.9)	−28.7 to 26.2	0.93
Water (g)	1504 (447)	1594 (483)	−344 to 165	0.48
Dietary fibre (g)	15.9 (5.69)	16.0 (6.33)	−3.45 to 3.13	0.92
Oligosaccharides (g)	2.65 (1.06)	2.79 (1.11)	−0.73 to 0.46	0.65
Polyols (g)	1.58 (1.72)	1.34 (1.07)	−0.55 to 1.02	0.54
Fructose in excess of glucose (g)	1.30 (0.80)	1.24 (0.72)	−0.36 to 0.47	0.78
lactose (g)	16.5 (8.61)	16.1 (7.31)	−3.95 to 4.78	0.85
Total FODMAPs with lactose (g)	22.0 (9.91)	21.4 (7.69)	−4.27 to 5.43	0.81
Total FODMAPs without lactose (g)	5.53 (2.70)	5.37 (2.11)	−1.16 to 1.49	0.80

Notes: ^a^ Two sample *t*-test.

**Table 5:** Major food sources of FODMAPs consumed by the 54 participants.

FODMAPs	Food Source	Contribution ^a^
Oligosaccharides	Nutritionals ^b^	17%
	Bread/toast	13%
	Cakes/slices	8%
	Wheat/oat cereals	6%
	Biscuits/crackers	6%
Polyols	Prunes	28%
	Canned pears ^c^	26%
	Canned peaches ^c^	9%
	Apple puree	7%
	Jellied fruit ^d^	2%
Fructose in excess of glucose	Canned pears ^c^	32%
	Fruit salad ^e^	12%
	Jellied fruit ^d^	9%
	Orange juice	6%
	Canned fruit salad ^f^	5%
lactose	Cow’s milk in hot beverages and on cereals	39%
	Porridge made with milk	27%
	Mousse	7%
	Yoghurt	6%
	Ice-cream	3%

Notes: ^a^ Contribution towards total oligosaccharides, polyols, fructose in excess of glucose or lactose consumed by 54 participants; ^b^ nutritional supplements containing fructo-oligosaccharides or galacto-oligosaccharides ^c^ FODMAPs estimated, fruit in syrup; ^d^ contains canned pears; ^e^ contains canned fruit salad in juice; ^f^ in fruit juice (apple or grape).

## Supplementary Materials

The following are available online at www.mdpi.com/2072-6643/9/10/1083/s1, Figure S1: Scatter plots with linear regressions of total FODMAP intake excluding lactose and various IBS symptom scores. Presented *R*-squared and *p*-values indicate the significance of the association between total FODMAP intake and IBS symptom score, Table S1: Baseline clinical features of 74 participants who completed GSRS-IBS questionnaire and 30 participants each selected for symptomatic and asymptomatic groups, Table S2: Number of symptomatic and asymptomatic participants reporting any discomfort and moderate to severe discomfort in the GSRS-IBS questionnaire.
