# Supplementary material for: Gastrointestinal Symptoms and FODMAP Intake of Aged-Care Residents from Christchurch, New Zealand
Source: Nutrients. 2017 Sep 29;9(10):1083. doi: 10.3390/nu9101083 (PMC5691700; doi:10.3390/nu9101083)
Supplement: Supplementary file 1 [file nutrients-09-01083-s001.zip › nutrients-216269-supplementary.pdf]

Table S1. Baseline clinical features of 74 participants who completed GSRS-IBS questionnaire and 30 participants each selected for symptomatic and asymptomatic groups.

Characteristic	74 Participants	Symptomatic Group (*n* = 30)	Asymptomatic Group (*n* = 30)
Body mass index [a]			
Underweight (<18.50 kg/m^2)	10 (14%)	2 (7%)	7 (23%)
Normal (18.50–24.99 kg/m^2)	37 (50%)	15 (50%)	14 (47%)
Overweight (25.00–29.99 kg/m^2)	21 (28%)	10 (33%)	7 (23%)
Obese (≥30.00 kg/m^2)	6 (8%)	3 (10%)	2 (7%)
Regular medications			
Treatment of constipation	38 (51%)	17 (57%)	13 (43%)
Treatment of diarrhoea	8 (11%)	5 (17%)	5 (17%)
Known to cause constipation	23 (31%)	10 (33%)	8 (27%)
Known to cause diarrhoea	5 (7%)	2 (7%)	3 (10%)
Comorbidities			
Cardiovascular	55 (74%)	22 (73%)	23 (77%)
Central nervous	39 (50%)	17 (57%)	16 (53%)
Respiratory	19 (26%)	7 (23%)	8 (27%)
Thyroid	8 (11%)	2 (7%)	4 (13%)
Musculoskeletal	42 (57%)	17 (57%)	17 (57%)
Diabetes	14 (19%)	6 (20%)	5 (17%)
Cognitive	25 (34%)	10 (33%)	9 (30%)
Psychiatric	17 (23%)	7 (23%)	7 (23%)
Cancer	22 (30%)	10 (33%)	6 (20%)
Lower GI disorder	15 (20%)	8 (27%)	5 (17%)
Function levels			
Continent of bowels	53 (72%)	21 (70%)	20 (67%)
Partial continence	5 (7%)	4 (13%)	0 (0%)
Incontinent	15 (20%)	4 (13%)	10 (33%)
Colostomy	1 (1%)	1 (3%)	0 (0%)
Able to mobilise	54 (73%)	21 (70%)	20 (67%)
Bed/chairbound	20 (27%)	9 (30%)	10 (33%)
Normal swallow	64 (86%)	25 (81%)	27 (90%)
Dysphagia (on pureed diet)	10 (14%)	5 (19%)	3 (10%)

Notes: [a] WHO age-independent BMI classification [25].

Table S2. Number of symptomatic and asymptomatic participants reporting any discomfort and moderate to severe discomfort in the GSRS-IBS questionnaire.

	Symptomatic Group (*n* = 30)		Asymptomatic Group (*n* = 30)	
**GSRS-IBS Subdomains and Individual Questions**	**Score ≥ 2 (Any Discomfort), *n***	**Score ≥ 4 (Moderate to Severe Discomfort), *n***	**Score ≥ 2 (Any Discomfort), *n***	**Score ≥ 4 (Moderate to Severe Discomfort), *n***
Pain (Q1 or 2)	15 (50%)	11 (37%)	1 (3%)	0
Q1 abdo. Pain	15	11	1	0
Q2 pain relieved by BM	8	3	0	0
Bloating (Q3, 4 or 13)	24 (80%)	3 (10%)	7 (23%)	1 (3%)
Q3 bloating	15	3	5	1
Q4 passing gas	16	2	4	0
Q13 abdo. distention	6	0	0	0
Constipation (Q5 or 8)	17 (57%)	8 (27%)	8 (27%)	3 (10%)
Q5 constipation	14	6	8	3
Q8 hard stools	14	6	1	0
Diarrhoea (Q6, 7, 9 or 10)	24 (80%)	10 (33%)	6 (20%)	0
Q6 diarrhoea	4	0	1	0
Q7 loose stools	16	4	5	0
Q9 urgency	8	6	3	0
Q10 incomplete BM	15	6	0	0
Satiety (Q11 or 12)	15 (50%)	3 (10%)	5 (17%)	2 (7%)
Q11 early satiety	11	2	4	2
Q12 prolonged satiety	8	2	1	0

Abbreviations: GSRS-IBS, IBS specific Gastrointestinal Symptom Rating Scale; abdo., abdominal; BM, bowel movement; GI, gastrointestinal. Notes: *n* (%) for each of six subdomains (highlighted) was determined by adding the number of participants who scored ≥2 or ≥4 for any of the relevant questions under each subdomain.

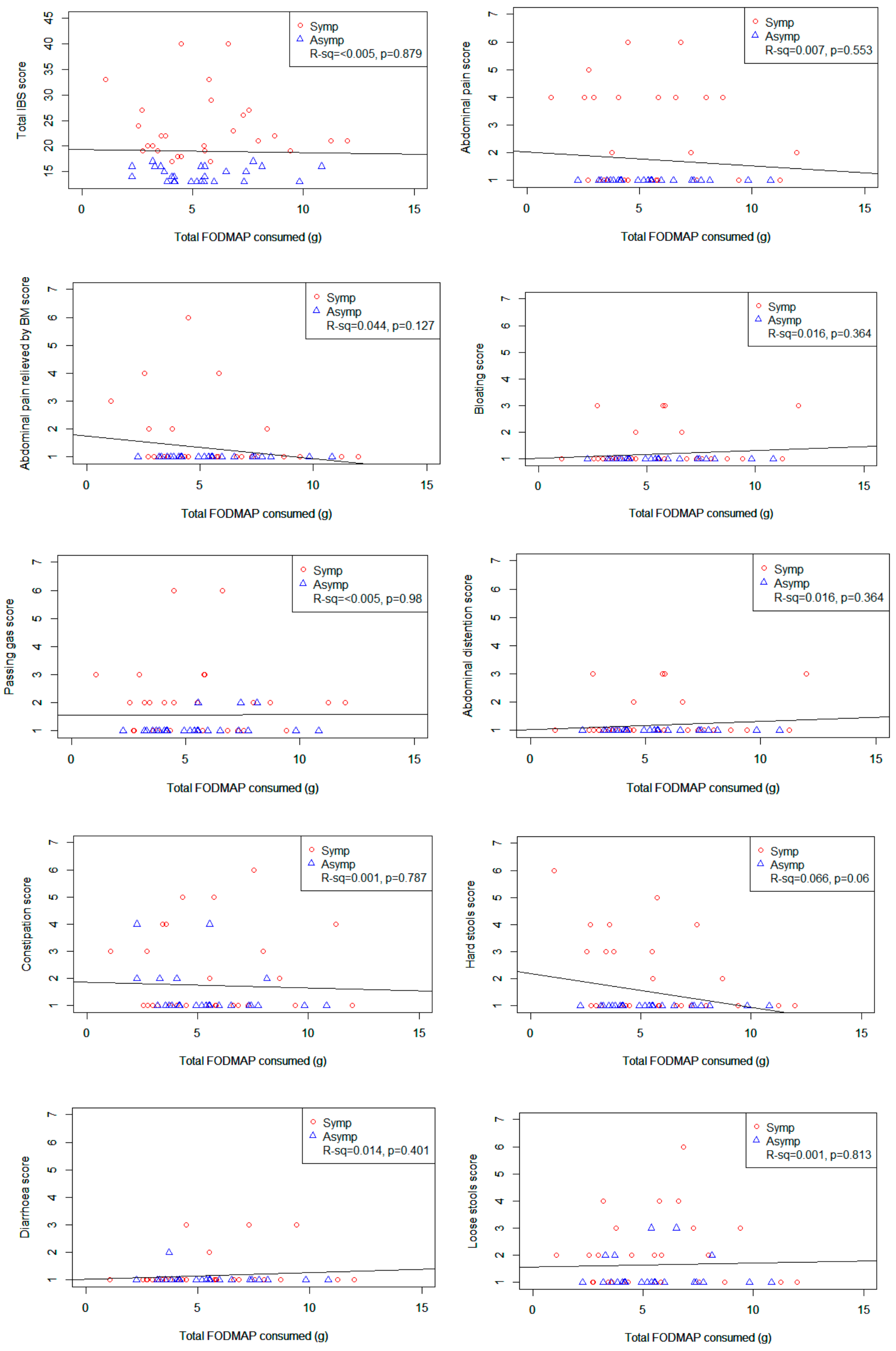

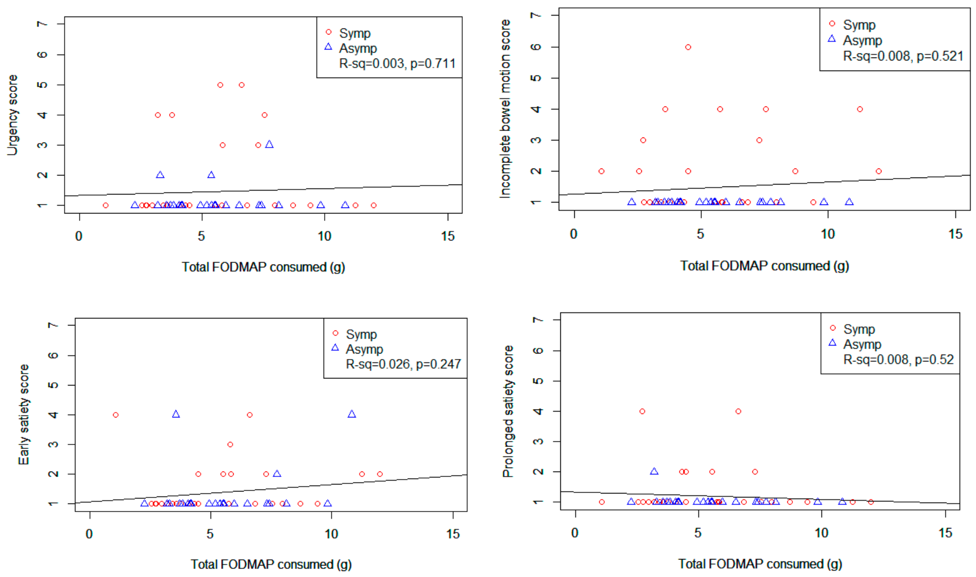

Figure S1. Scatter plots with linear regressions of total FODMAP intake excluding lactose and various IBS symptom scores. Presented R-squared and *p*-values indicate the significance of the association between total FODMAP intake and IBS symptom score.
